# Determinants of Outcome Variability in Ischemic Stroke: A Focus on Routinely Collected Biomarkers

**DOI:** 10.3390/antiox14111305

**Published:** 2025-10-30

**Authors:** Alexandru Gerdanovics, Sorana D. Bolboacă, Ioana Cristina Stănescu, Camelia Manuela Mîrza, Gabriela Bombonica Dogaru, Cristina Ariadna Nicula, Paul Mihai Boarescu, Cezara-Andreea Gerdanovics, Adriana-Elena Bulboacă

**Affiliations:** 1 Department of Pathophysiology, “Iuliu Haţieganu” University of Medicine and Pharmacy, Victor Babeş Street, No. 2–4, 400012 Cluj-Napoca, Romania; alexandru.gerdanovics@elearn.umfcluj.ro (A.G.);; 2 Clinical Rehabilitation Hospital, Viilor Street, No. 46–50, 400066 Cluj-Napoca, Romania or ioanastane@elearn.umfcluj.ro (I.C.S.);; 3 Department of Medical Informatics and Biostatistics, “Iuliu Haţieganu” University of Medicine and Pharmacy, Louis Pasteur Street, No. 6, 400349 Cluj-Napoca, Romania; 4 Department of Neurology, “Iuliu Hațieganu” University of Medicine and Pharmacy Cluj-Napoca, Victor Babeş Street, No. 43, 400012 Cluj-Napoca, Romania; 5 Department of Balneophysiokinetotherapy and Medical Recovery, Faculty of Nursing and Health Sciences, “Iuliu Hațieganu” University of Medicine and Pharmacy Cluj-Napoca, Victor Babeş Street, No. 8, 400347 Cluj-Napoca, Romania; 6 Department of Oro-Maxilo Facial Surgery and Radiology, Faculty of Dental Medicine, “Iuliu Haţieganu” University of Medicine and Pharmacy, Victor Babeş Street, No. 2–4, 400012 Cluj-Napoca, Romania; 7 County Emergency Clinical Hospital, Clinicilor Street, No. 3–5, 400347 Cluj-Napoca, Romania; 8 Department of Biomedical Sciences, Faculty of Medicine and Biological Sciences, “Ștefan cel Mare” University of Suceava, Universităţii Street, No. 13, 720229 Suceava, Romania; paul.boarescu@usm.ro; 9 Department of Internal Medicine, 4th Medical Discipline, “Iuliu Hațieganu” University of Medicine and Pharmacy, Republicii Street, No. 18, 400015 Cluj-Napoca, Romania; andreea.ceza.irimie@elearn.umfcluj.ro; 10 Oncology Institute “Prof. Dr. Ion Chiricuță”, Republicii Street, No. 34–36, 400015 Cluj-Napoca, Romania

**Keywords:** stroke, ischemic, neuroinflammation, oxidative, stress, metabolic, factors

## Abstract

Ischemic stroke remains a leading cause of mortality and disability, with proinflammatory, metabolic, and oxidative stress-related factors contributing to outcome variability. We conducted a retrospective cross-sectional study of 124 consecutive patients (53 women, 71 men; median age 71 [62–76]) discharged with ICD-10 code I69.3 from the Neurology Department of the Clinical Rehabilitation Hospital in Cluj-Napoca (January 2023–September 2024). Men were younger (median age of 69 vs. 73 years, *p*-value = 0.010), more frequently smokers (42% vs. 9%, *p* < 0.001), and alcohol consumers (21% vs. 4%, *p*-value = 0.007) than women. In contrast, women were more frequently sedentary (68% vs. 49%, *p*-value = 0.038) and had higher LDL cholesterol (89 vs. 74 mg/dL, *p* = 0.026) than men. Patients with at least moderate disability (n = 84) presented higher levels of C-Reactive Protein (CRP), 1.4 vs. 1.1 mg/L, *p*-value = 0.027) and more frequently low HDL cholesterol serum levels (29.8% vs. 7.5%, *p*-value = 0.006) compared to those with minor disability. In multivariable regression, low HDL was the sole independent predictor of disability severity (OR = 4.58, 95% CI 1.21–17.41; AUC = 0.78, sensitivity = 88%, specificity = 42%), while CRP and age did not retain the significance obtained in univariable regression. Our findings highlight sex-specific risk profiles and underline the contribution of proinflammatory, metabolic, and oxidative pathways to ischemic stroke severity, underscoring the need for prospective validation in larger cohorts.

## 1. Introduction

Oxidative stress is defined as an imbalance between the excessive generation of reactive oxygen species (ROS) and reactive nitrogen species (RNS) and the antioxidant defense mechanisms that counteract them [1]. Mitochondria, which produce nearly 90% of cellular ATP and regulate calcium homeostasis and thermogenesis, are a major source of ROS [1]. Under mitochondrial dysfunction, excessive ROS and RNS accumulate, causing oxidative damage of lipids, proteins, and DNA (deoxyribonucleic acid). Free radicals may arise from exogenous stressors such as air pollution or smoking, or from endogenous metabolic processes when antioxidant defenses are overwhelmed [2]. At physiological levels, ROS act as secondary messengers in signaling pathways regulating proliferation, differentiation, and apoptosis [3]. However, excessive or mislocalized ROS production disrupts homeostasis and contributes to chronic degenerative and cardiovascular diseases through cumulative biomolecular damage [3].

The brain is particularly vulnerable to oxidative stress due to its high metabolic rate and oxygen demand. During ischemic stroke, reduced cerebral blood flow deprives neurons of oxygen and glucose, leading to mitochondrial dysfunction, ROS overproduction, excitotoxicity, and neuroinflammation, ultimately exacerbating neuronal injury and blood–brain barrier disruption [1,4]. In the subacute and chronic phases, persistent oxidative stress—together with systemic risk factors such as dyslipidemia, diabetes, and smoking—sustains vascular dysfunction and impairs recovery, underscoring the central role of oxidative pathways in stroke pathophysiology [5].

Research has recently focused on integrating inflammatory, metabolic, and oxidative pathways into a unified model of cerebrovascular injury. Biomarkers such as high-sensitivity C-reactive protein (hs-CRP), oxidized low-density lipoproteins (ox-LDL), and interleukin-6 (IL-6) have been shown to predict poor outcomes and recurrent events, reflecting a state of persistent low-grade inflammation that bridges metabolic and vascular dysfunction [5,6]. Moreover, studies establish the role of adipokines, such as leptin and adiponectin, and oxidative enzymes including myeloperoxidase and paraoxonase-1, in modulating endothelial reactivity and plaque stability [7]. Understanding the interplay between these molecular mediators and classical risk factors such as hypertension and diabetes will help refine patient stratification and open up new therapeutic perspectives targeting redox balance and vascular repair mechanisms.

Stroke remains the second leading cause of mortality and the third leading cause of disability worldwide [8]. Ischemic stroke, together with acute myocardial infarction, accounts for nearly 85% of cardiovascular disease-related deaths [9]. In Europe, the prevalence is highest in Eastern regions, with Romania reporting an incidence of 262 cases per 100,000 inhabitants and 38,725 hospital admissions in 2022, resulting in an in-hospital mortality rate of 12% [10]. According to the TOAST classification, ischemic stroke is divided into five categories, of which large-artery atherosclerosis and cardioembolism are the most frequent [11]. Atherogenesis, driven by hypertension, dyslipidemia, smoking, and diabetes, involves endothelial injury, lipid accumulation, inflammation, and plaque rupture, followed by thrombus formation and arterial occlusion [12,13,14]. These processes trigger excitotoxicity, oxidative stress, and inflammation, collectively defined as neuroinflammation [15,16].

Neuroinflammation extends beyond the acute stage, persisting into the subacute and chronic phases, where it maintains oxidative stress, promotes vascular dysfunction, and drives secondary neuronal damage [11,17,18]. This prolonged inflammatory–metabolic state is shaped by both modifiable (e.g., hypertension, diabetes, dyslipidemia, smoking, obesity, sedentary lifestyle, hyperhomocysteinemia, periodontal disease) and non-modifiable (e.g., age, sex, race, genetics) risk factors [11]. In Romania, stroke imposes a substantial societal burden, with average first-year costs of EUR 5227 per patient, more than 80% being direct medical expenses [19]. Aphasia-related costs exceed EUR 3 million annually [20], and between 2007 and 2015, stroke incidence nearly doubled, with yearly expenditures surpassing EUR 650 million [21]. Beyond these economic aspects, Romanian studies highlighted genetic and metabolic determinants: a case–control study found no significant association between the TNFα-308G>A polymorphism and ischemic stroke [22], while a prospective analysis linked lipid profile abnormalities, particularly low HDL cholesterol, with worse outcomes [23]. In this context, our study focused on circulating inflammatory and lipid markers as potential modulators of post-stroke outcomes.

Building on previous evidence, our study focuses on inflammatory and metabolic pathways as central drivers of sustained neuroinflammation and variability in ischemic stroke outcomes. We hypothesized that distinct patterns would emerge according to sex and level of disability. By examining these determinants in patients hospitalized for rehabilitation in the Neurology Department of the Clinical Rehabilitation Hospital in Cluj-Napoca, we aimed to provide an exploratory analysis of their contribution to stroke severity and recovery.

## 2. Materials and Methods

The study adhered to the Helsinki Declaration and had received approval from the Ethics Committee of the Scientific Research Ethics Commission of the “Iuliu-Haţieganu” University of Medicine and Pharmacy, Cluj-Napoca (Approval number 37/2 April 2025). The Scientific Research Ethics Commission of the Cluj-Napoca Clinical Recovery Hospital also approved the study (Approval number 20/23 October 2024). The ethics committees approved a waiver of consent since the study used only de-identified, historical medical data.

### 2.1. Study Design and Setting

We conducted an observational, retrospective, cross-sectional study at the Cluj-Napoca, Clinical Rehabilitation Hospital, including patients admitted to the Neurology Department between January 2023 to September 2024.

### 2.2. Participants

Patients were selected based on the diagnosis of ischemic stroke (ICD 10-I69.3—Sequelae of cerebral infarction). Although no formal local validation has been conducted in Romania, international studies have reported good accuracy for this code, with positive predictive values generally above 80% and sensitivity reaching over 90% when used as the principal diagnosis [24,25]. All cases were documented with neurologic examination, using National Institutes of Health Stroke Scale (NIHSS ≥ 0), assessed at the time of hospital admission, recorded by the attending neurologist [26], and imagistic criteria (native or angio-cranial Computer Tomography—ruling out hemorrhage or tumors) [27]. Patients hospitalized for other neurological pathologies, without a diagnosis of ischemic stroke documented by imaging, septic, or with any known inflammatory disease in acute flare-up, were excluded. Patient’s neurological and functional status at the time of transfer to the rehabilitation department, corresponding to the subacute phase after ischemic stroke, was evaluated by NIHSS score.

### 2.3. Data Extraction and Preparation

Medical records and the computer data system of the hospital, “Atlas Med” were the source of our data. Patients were selected using a filter based on the primary diagnosis at discharge (ICD-I69.3). Subsequently, from the patients’ follow-up sheets, we obtained data on hereditary collateral history (HCA), medical history, consumption of toxic substances, clinical examination, and laboratory tests. Biomarkers were measured at the time of admission to the hospital following the same protocol. The eligible patients were those admitted for neurological rehabilitation after an ischemic stroke, so, the timing from the acute event to the admission varied depending on the patient’s functional prognosis and transfer logistics. Information on the presence of comorbidities (e.g., hypertension, diabetes, dyslipidemia, chronic kidney disease) was collected from the medical charts; diagnoses were established based on clinical records and laboratory criteria as described above. Information regarding chronic medication use, including statin therapy, was inconsistently reported in hospital charts and, therefore, not included in the present analysis.

**Figure 1:**
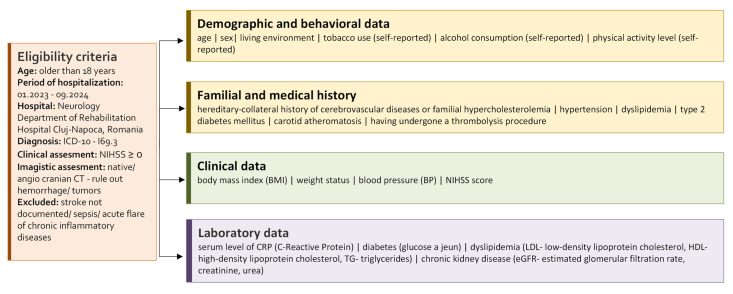
Baseline characteristics and clinical data of the study cohort.

To reduce potential bias, we included all consecutive patients with ICD-10 I69.3 discharge diagnosis during the study period, confirmed by both clinical and imaging criteria. Data was extracted from a single hospital electronic system to minimize information bias (Figure 1). Potential confounding was addressed in the statistical analysis.

The study size was determined by the number of consecutive patients meeting inclusion criteria during the study period, without a priori sample size calculation.

Concerning the laboratory examinations, we evaluated the presence of diabetes mellitus type 2 (*a-jeun* blood glucose > 126 mg/dL), dyslipidemia (LDL-cholesterol > 100 mg/dL or HDL-cholesterol < 40 mg/dL or triglycerides (TG) > 150 mg/dL), chronic kidney disease (estimated glomerular filtration rate—eGFR < 60 mL/min/1.73 m^2^). Estimated glomerular filtration rate (eGFR) was calculated using CKD-EPI Formula [28]. Border values for evaluation were obtained from several guidelines [29,30,31,32]. These markers were also considered relevant due to their previously reported associations with systemic inflammation, oxidative stress, and vascular dysfunction in ischemic stroke.

### 2.4. Data Analysis

Primary data were obtained from the electronic system (Atlas Med) and patients’ charts. Data cleaning involved verification of the extracted variables against the original patient records to ensure accuracy. Given the exploratory nature of this study, particular attention was paid to markers reflecting inflammatory and oxidative stress-related pathways (e.g., CRP, HDL). Data was collected in Microsoft Excel and processed in search of different associations between specific groups, sex, and level of disability (NIHSS categories). Continuous variables are reported as median [first quartile (Q1) to third quartile (Q3)], and comparisons between groups were performed using the Mann–Whitney U test according to the evaluation of distribution by groups. Categorical variables are reported as counts and percentages, and differences between groups were assessed using the Chi-squared test or Fisher’s exact test, as appropriate. Bivariate associations were examined using Spearman’s rank correlation coefficient. Univariable logistic regression analyses were conducted to evaluate the association between potential predictors and the outcome of interest. Variables with a *p*-value < 0.10 in univariable analyses were entered into forward stepwise multivariable logistic regression models to adjust for potential confounders. Results are presented as odds ratios (ORs) with 95% confidence intervals (CIs). Considering an exploratory approach, a two-tailed *p*-value < 0.05 was considered statistically significant. Statistical analyses were performed using Jamovi [version 2.6.26.0].

## 3. Results

**Figure 2:**
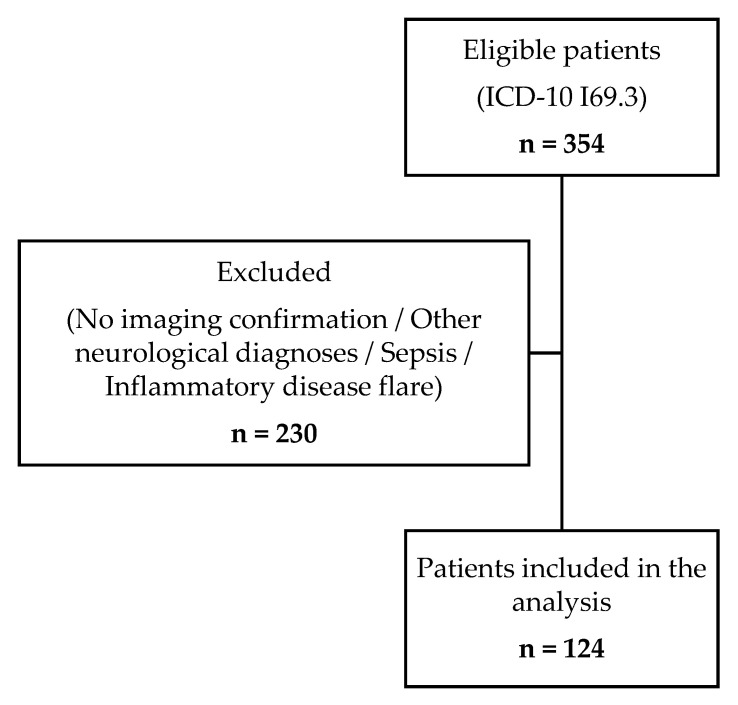
Flow diagram of patient selection process.

Our cohort consisted of 124 patients, aged from 24 to 94, with a high proportion of men (57.3%). Subjects’ selection process is illustrated in Figure 2. Half of the cohort were overweight or obese, with the presence of carotid atherosclerosis observed in three-quarters of participants (Table 1).

### 3.1. Patients’ Characteristics by Sex

Most of the evaluated patients were male, with statistically significant differences between men and women (Table 1). Only nine patients received embolization treatment, 5/53 women and 4/71 men (Fisher’s exact test: *p*-value = 0.4949). Men and women had similar characteristics except for age (younger men), smoking and self-declared alcohol consumption status (higher frequency among men), sedentarism (higher frequency among women), higher serum levels of LDL in women, and lower HDL serum levels in men, and higher levels of creatinine in men (Table 1).

Most participants had LDL levels in the normal range, with similar frequencies of higher levels (7.5% in women and 7.0% in men), but the frequency of men with lower LDL levels was higher (28.2%) than in women (9.4%) (Fisher’s exact test: *p*-value = 0.0320). Lower serum levels of HDL were similar among men (23.9%) and women (18.9%) (Fisher’s exact test: *p*-value = 0.7230). A higher frequency of men showed higher values of serum creatinine levels (21.1%) than women (9.4%), while lower levels were more frequently seen in women (9.4%) than men (2.8%), but the differences only reach a tendency to statistical significance (Fisher’s exact test: *p*-value = 0.092).

### 3.2. Patients’ Characteristics by Disability Class

Eighty-four patients had a NIHSS score of at least moderate, with no patient classified with a severe deficit. Thrombolysis was applied in one patient with a mild disability score and in eight patients with at least a moderate disability score. Patients with a mild disability score were older, had a lower frequency of atheromatosis, lower serum values of CRP and eGFR, and a higher serum creatinine level (Table 2).

Lacunarism is more frequent among patients with minor disability (62.5%) than those with at least moderate disability (47.6%), but the difference did not reach the significance threshold (Chi-squared test: *p*-value = 0.1209). Patients with at least a moderate disability had more frequently lower levels of HDL cholesterol (29.8%) than patients with minor disability (7.5%), the difference being statistically significant (Fisher’s exact test: *p*-value = 0.0056).

Among characteristics that showed statistically significant differences between groups, the abnormal value of HDL cholesterol proved the strongest predictor in univariable regression analysis (Table 3). The presence of abnormal values of HDL remains also in the multivariable logistic regression analysis the most important factor, but the model can correctly classify 88.1% of patients with at least moderate disability and 42.5% of those with minor disability (Acc—accuracy = 73%, Se—sensibility = 88%, Sp—specificity = 42%, AUC—area under the curve = 0.78). Inclusion of eGFR in the multivariable model led to only HDL class as a statistically significant predictor.

### 3.3. Bivariate Association of Quantitative Factors

**Figure 3:**
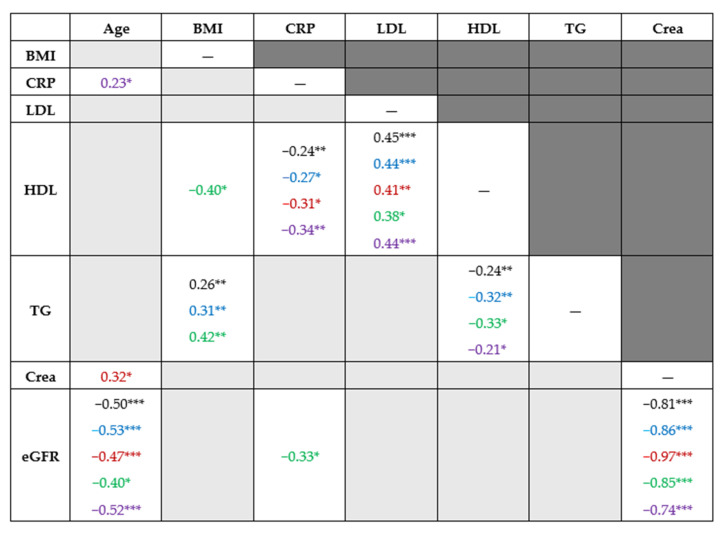
Bivariate association of quantitative factors. Note. * *p*-value < 0.05, ** *p*-value < 0.01, *** *p*-value < 0.001. Light gray background indicates the absence of statistically significant correlations. The reported values represent Spearman’s rank correlation coefficients. Blue indicates the values for men, red for women, green for patients with minor disability and violet for patients with at least a moderate disability. BMI—body mass index; CRP—C-reactive protein, LDL—low-density lipoprotein cholesterol, HDL—high-density lipoprotein cholesterol, TG—triglycerides, CRE—creatinine, eGFR—estimated glomerular filtration rate.

Age showed a statistically significant association with eGFR, eGFR with serum level of creatinine, HDL with CRP, and LDL and TG with BMI and HDL cholesterol (Figure 3).

Similar monotonic association was observed in the whole cohort (Spearman’s rank correlation coefficients in black), in men (blue), in women (red), in patients with minor disability (green), and those with at least moderate disability (violet) (Figure 3).

## 4. Discussions

### 4.1. Key Results

Our findings partially support the initial hypothesis by highlighting distinct patterns of demographic and proinflammatory risk factors within ischemic stroke patients and their association with disability severity. Men were younger and more frequently auto-reported tobacco and alcohol consumption, whereas women were more sedentary and had higher LDL cholesterol levels (Table 1). Patients with more severe disability presented higher CRP values and a greater prevalence of low HDL cholesterol, while those with minor disability tended to be older and showed reduced renal function (Table 2). In multivariable regression, low HDL cholesterol emerged as the sole independent predictor of disability severity (Table 3). Together with elevated CRP levels, this profile reflects not only systemic inflammation but also an altered oxidative balance, reinforcing the concept of oxidative stress as a key determinant of stroke severity and recovery [33,34].

### 4.2. Interpretation

Beyond its biochemical implications, the observed pattern supports the hypothesis of a crosslink between systemic metabolic disorders and localized neurovascular injury of that oxidative stress. Experimental studies demonstrated that excess ROS could alter the permeability of the blood–brain barrier and activate microglia, perpetuating neuronal apoptosis and white matter damage [35,36]. Furthermore, metabolic conditions such as diabetes and dyslipidemia amplify this oxidative burden through mitochondrial dysfunction and lipid peroxidation [37], leading to a self-sustaining proinflammatory environment. This integrative framework aligns with recent translational models that emphasize “metabolic inflammation” as a key driver of post-stroke neurodegeneration and reduced functional recovery.

Sex-related differences in vascular risk factors have been documented in stroke populations [38,39,40]. Our findings are consistent with studies showing that men are more frequently exposed to behavioral risks such as tobacco and alcohol consumption, whereas women often present with metabolic abnormalities, including higher LDL cholesterol (Table 1). These patterns may contribute to sex-specific differences in stroke incidence, severity, and recovery reported across various cohorts.

The independent association between low HDL cholesterol and disability severity underscores the potential role of lipid metabolism in post-stroke outcomes (Table 3). Prior studies have highlighted the anti-inflammatory, antioxidant, and vasoprotective effects of HDL [41,42], and our findings are in line with this evidence. Beyond lipid transport, HDL neutralizes oxidized LDL, reduces lipid peroxidation, and protects endothelial nitric oxide availability, thereby counteracting oxidative stress and vascular dysfunction [42]. Elevated CRP levels in patients with more severe disability (Table 2) emphasize the contribution of systemic inflammation and oxidative stress to stroke severity, as CRP can activate NADPH oxidase, enhance ROS production, and impair endothelial nitric oxide signaling, thereby amplifying neuroinflammation [6]. While rare, inborn errors of immunity have been associated with vascular inflammation and thrombotic complications, potentially predisposing to ischemic stroke in selected patients [43]. These mechanisms, however, were not investigated in the present cohort and warrant further dedicated research.

Although our dataset did not include information on statin therapy, we acknowledge that statin therapy may influence both lipid fractions and inflammatory profiles. Evidence indicates that statin treatment improves outcomes in specific subtypes of ischemic stroke, such as Embolic Stroke of Undetermined Source (ESUS), a well-defined form of cryptogenic stroke characterized by prominent inflammatory and endothelial dysfunction mechanisms [44]. These findings reinforce the importance of integrating medication history and stroke subtype data in future studies exploring inflammatory and oxidative mechanisms. Renal dysfunction is an established predictor of adverse outcomes after ischemic stroke, with several studies demonstrating associations between reduced eGFR, higher creatinine levels, and greater disability or mortality [45,46]. In our cohort, impaired renal function was paradoxically more common among patients with minor disability. This discrepancy may reflect selection bias, survival effects, or residual confounding inherent to retrospective designs (Table 2). Impaired renal function is associated with increased oxidative stress due to reduced clearance of reactive metabolites and accumulation of uremic toxins, which may further aggravate vascular injury and compromise neurological recovery [47]. Nevertheless, the integration of renal markers with lipid and inflammatory parameters highlights the complex interplay of metabolic and systemic factors in ischemic stroke, supporting the need for multifactorial risk assessment.

Although low HDL cholesterol remained the sole independent predictor of disability severity in the multivariable model (Table 3), its clinical utility appears limited. The model achieved good sensitivity but poor specificity, correctly classifying most patients with more severe disability but failing to discriminate those with minor deficits. This imbalance suggests that HDL alone cannot serve as a reliable prognostic biomarker in clinical practice. Rather, it may be better interpreted as part of a broader inflammatory–metabolic profile. At the same time, the paradoxical finding regarding renal function underlines the need for cautious interpretation and further validation in larger, prospective studies.

By providing evidence from a Romanian stroke cohort, our study highlights the importance of further research to validate these associations and to develop multifactorial prognostic models with clinical relevance.

### 4.3. Generalizability

Our findings should be interpreted with caution regarding external validity. The analysis was conducted in a single rehabilitation hospital in Romania, and the demographic and clinical characteristics of the included patients may not fully reflect ischemic stroke populations in other regions or healthcare systems. The observed sex-related differences and the association of low HDL cholesterol with disability severity are consistent with international evidence, suggesting that these patterns may be broadly applicable. In contrast, the findings regarding renal function diverge from prior studies, indicating that local factors, study design, or sample characteristics may have influenced the results. Confirmation in larger, multicenter, and prospective cohorts is therefore required to establish the extent to which our results can be generalized.

### 4.4. Limitations

We acknowledge several limitations in our study. The applied study design as retrospective and cross-sectional study of routinely collected healthcare data limits the possibility to establish causal relationships of the reported patterns. The absence of follow-up data further restricts the evaluation of long-term outcomes. The analysis was conducted in a single rehabilitation hospital, which may not fully capture the variability of ischemic stroke populations in other clinical or geographic contexts. Consequently, the external validity of the findings is limited.

The source of data was represented by the routinely collected hospital data. In this context, certain variables, such as tobacco and alcohol consumption, were self-reported and may be subject to social desirability and acquiescence bias. The absence of data regarding statin use, not routinely collected, is a major limitation of our study. Statin therapy can modulate both lipid profiles and inflammatory responses, factors that are closely linked to stroke outcomes. Therefore, not accounting for this variable may have introduced confounding effects affecting the associations observed between HDL cholesterol, CRP, and disability severity as stroke outcome.

Another limitation is that most patients included in our study did not undergo thrombolytic therapy. Consequently, our results mainly reflect risk factor patterns and outcomes in non-thrombolyzed ischemic stroke patients. It remains uncertain whether patients treated with thrombolysis might present distinct inflammatory, metabolic, or oxidative profiles, and further studies comparing thrombolyzed versus non-thrombolyzed cohorts are warranted.

The absence of a control group restricts the possibility of comparing the prevalence of behavioral and metabolic risk factors in our cohort with those observed in the general population. For instance, smoking and alcohol consumption patterns reported in Table 1 cannot be directly contextualized outside the evaluated sample.

Only a limited set of biomarkers was assessed, focusing primarily on CRP and lipid profile parameters. We did not include direct markers of oxidative stress (e.g., malondialdehyde, 8-iso-PGF2α, glutathione, superoxide dismutase, catalase), which limits our ability to capture the redox imbalance associated with ischemic stroke. Future prospective studies integrating oxidative stress and cytokine biomarkers, along with imaging markers of vascular dysfunction, may further clarify causal pathways.

As shown in Table 3, HDL cholesterol emerged as an independent predictor of disability severity; however, the lack of additional inflammatory and metabolic markers (e.g., cytokines, adipokines, genetic factors) may have introduced residual confounding. Furthermore, although low HDL cholesterol emerged as a statistically significant predictor in the multivariable model (Table 3), the modest specificity observed limits its immediate clinical applicability. Moreover, the association between low HDL cholesterol and disability severity (Table 3) might also be partially explained by lifestyle factors such as diet and physical activity. As shown in Table 1, women were significantly more sedentary than men, and sedentarism itself is a known determinant of reduced HDL levels, which could have contributed to the observed differences.

## 5. Conclusions

In our retrospective cross-sectional study, we identified distinct demographic and biochemical patterns among ischemic stroke patients during rehabilitation. Sex-related differences were evident, with men more frequently reporting behavioral risk factors and women presenting metabolic imbalances. Among biochemical markers, low HDL cholesterol remained the sole independent predictor of disability severity, although its clinical utility appeared limited due to modest specificity. The results underline the possible contribution of metabolic, inflammatory, and oxidative pathways to functional outcomes in ischemic stroke, highlighting the need for integrated risk factor management and for prospective studies including redox biomarkers to validate the observed associations.

## Figures and Tables

**Table 1:** Patients’ characteristics by sex.

	All, n = 124	Women, n = 53	Men, n = 71	*p*-Value
Demographics and Lifestyle Factors				
Age, years ^a^	71 [62 to 76.3]	73 [67 to 77]	69 [57 to 75]	0.0098
Rural living ^b^	48 (38.7)	23 (43.4)	25 (35.2)	0.3546
Smoker ^b^	35 (28.2)	5 (9.4)	30 (42.3)	<0.0001
Alcohol consumption ^b^	17 (13.7)	2 (3.8)	15 (21.1)	0.007
Sedentarism ^b^	71 (57.3)	36 (67.9)	35 (49.3)	0.038
Comorbidities				
BMI, kg/m^2 a^	27.5 [24.6 to 31.2]	28.1 [24.9 to 32.8]	27.4 [24.1 to 30.9]	0.3052
Obesity ^b^	46 (37.1)	23 (43.4)	23 (32.4)	0.2096
Type II Diabetes Mellitus ^b^	37 (29.8)	13 (24.5)	24 (33.8)	0.2642
Hypertension ^b^	57 (46)	24 (45.3)	33 (46.5)	0.8948
Clinical atherosclerotic signs ^b^	22 (17.7)	7 (13.2)	15 (21.1)	0.2535
Carotid atherosclerosis ^b^	93 (75)	39 (73.6)	54 (76.1)	0.7532
Kidney chronic disease ^b^	20 (16.1)	10 (18.9)	10 (14.1)	0.4737
AF ^b^	34 (27.4)	14 (26.4)	20 (28.2)	0.8285
HCA (CVD/FHC) ^b^	17 (13.7)	7 (13.2)	10 (14.1)	>0.9999
Biomarkers				
CRP ^a^	1.2 [0.7 to 2.3]	1.3 [0.9 to 2.4]	1.1 [0.6 to 1.9]	0.1577
LDL ^a^	77.5 [63 to 108.3]	89 [69 to 111]	74 [59 to 106.5]	0.0261
HDL ^a^	44 [35 to 54.3]	47 [37 to 56]	39 [35 to 51.5]	0.0526
TG ^a^	127.5 [94 to 164.5]	122 [94 to 154]	135 [94.5 to 169]	0.8143
Creatinine ^b^	0.8 [0.7 to 1.1]	0.8 [0.6 to 0.9]	0.9 [0.8 to 1.2]	0.0022
eGFR ^a^	83.7 [61.4 to 96]	78.9 [59.3 to 93.5]	86 [64.8 to 98.7]	0.0627
Disability Score				
NIHSS ^a^	6 [3.75 to 10]	7 [3 to 10]	6 [4 to 10]	0.8289
NIHSS class ^b^ minor	34 (27.4)	14 (26.4)	20 (28.2)	0.9645
moderate	80 (64.5)	35 (66)	45 (63.4)	
moderate to severe	4 (3.2)	2 (3.8)	2 (2.8)	

^a^ data are reported as median [Q1 to Q3], where Q is the value of the quartile; comparison made with Mann–Whitney test; ^b^ data are reported as no. (%), and differences between women and men are tested with Chi-squared test or Fisher’s exact test; BMI—body mass index; AF—atrial fibrillation, HCA—hereditary-collateral history, CVD—cardiovascular diseases, FHC—familial hypercholesterolemia, CRP—C-reactive protein, LDL—low-density lipoprotein cholesterol, HDL—high-density lipoprotein cholesterol, TG—triglycerides, eGFR—estimated glomerular filtration rate.

**Table 2:** Patients’ characteristics by different disability class: minor vs. at least moderate.

	NIHSS Minor, n = 40	NIHSS at Least Moderate, n = 84	*p*-Value
Demographics			
Age, years ^a^	74.5 [63.8 to 79]	69 [60.8 to 75]	0.0148
Rural living ^b^	13 (32.5)	16 (19)	0.0981
Smoker ^b^	14 (35)	21 (25)	0.2475
Alcohol consumption ^b^	5 (12.5)	12 (14.3)	>0.9999
Sedentarism ^b^	20 (50)	51 (60.7)	0.2596
Comorbidities			
BMI, kg/m^2 a^	28.8 (5.8)	27.8 (5.1)	0.3463
Obesity ^b^	17 (42.5)	29 (34.5)	0.3901
Type II Diabetes Mellitus ^b^	13 (32.5)	24 (28.6)	0.6549
Hypertension ^b^	19 (47.5)	38 (45.2)	0.8132
Clinical atherosclerotic signs ^b^	29 (72.5)	73 (86.9)	0.0497
Carotid atherosclerosis ^b^	29 (72.5)	64 (76.2)	0.6573
Chronic kidney disease ^b^	13 (32.5)	16 (19)	0.0981
AF ^b^	8 (20)	26 (31)	0.2013
HCA (CVD/FHC) ^b^	6 (15)	11 (13.1)	0.7731
Biomarkers			
CRP ^a^	1.1 [0.6 to 1.4]	1.4 [0.8 to 2.5]	0.0273
LDL ^a^	79 [66 to 102.8]	77 [61.5 to 109.3]	0.7769
HDL ^a^	46.5 [36.8 to 57]	43.5 [33 to 52]	0.1830
TG ^a^	135 [100 to 168]	124.5 [87 to 160.3]	0.5764
Creatinine ^b^	0.9 [0.7 to 1.2]	0.8 [0.7 to 1]	0.0066
eGFR ^a^	66.3 [57.6 to 85.5]	88.4 [70.5 to 98.6]	0.0011

^a^ data are reported as median [Q1 to Q3], where Q is the value of the quartile; comparison made with Mann–Whitney test; ^b^ data are reported as no. (%), and differences between women and men are tested with Chi-squared test or Fisher’s exact test; BMI—body mass index; AF—atrial fibrillation, HCA—hereditary-collateral history, CVD—cardiovascular diseases, FHC—familial hypercholesterolemia, CRP—C-reactive protein, LDL—low-density lipoprotein cholesterol, HDL—high-density lipoprotein cholesterol, TG—triglycerides, eGFR—estimated glomerular filtration rate.

**Table 3:** Logistic regression analysis: risk factors for association with disability level.

	Univariable Regression				Multivariable Regression *		
	Equation	AIC	*p*-Value	OR [95%CI]	B (SE)	*p*-Value	OR [95%CI]
Intercept					4.71 (1.60)	0.0033	
Age, years	3.85 − 0.04 × Age	154.3	5.67 (0.0173)	0.96 [0.92 to 0.99]	−0.05 (0.02)	0.0365	0.96 [0.92 to 1.00]
CRP	0.23 + 0.31 × CRP	153.0	6.90 (0.0086)	1.37 [1.00 to 1.87]	0.31 (0.17)	0.0700	1.36 [0.98 to 1.89]
CRE	2.42 − 1.85 × CRE	152.8	7.19 (0.0073)	0.16 [0.04 to 0.64]	−1.73 (0.79)	0.0290	0.18 [0.04 to 0.84]
eGFR	−1.70 + 0.03 × eGRF	148.7	11.22 (0.0008)	1.03 [1.01 to 1.05]			
HDL class	0.47 − 1.65 × HDL	151.1	8.88 (0.0029)	5.23 [1.47 to 18.54]	1.52 (0.68)	0.0253	4.58 [1.21 to 17.41]

* AIC = 140.9, χ^2^ (*p*-value) = 25.02 (<0.0001); CRP—C-reactive protein, CRE—creatinine, eGFR—estimated glomerular filtration rate, HDL—high-density lipoprotein cholesterol.

## Data Availability

The data presented in this study are available on reasonable request from the corresponding author. The data is not publicly available due to privacy and ethical restrictions.

## Abbreviations

The following abbreviations are used in this manuscript:

Acc	Accuracy
AF	Atrial Fibrillation
ATP	Adenosine–triphosphate
AUC	Area Under the Curve
BMI	Body Mass Index
CI	Confidence Interval
CKD-EPI	Chronic Kidney Disease Epidemiology Collaboration (formula)
CRP	C-Reactive Protein
CT	Computed Tomography
CVD	Cardiovascular Diseases
eGFR	Estimated Glomerular Filtration Rate
ESUS	Embolic Stroke of Undetermined Source
FHC	Familial Hypercholesterolemia
HCA	Hereditary-Collateral History
HDL	High-Density Lipoprotein Cholesterol
ICD-10	International Classification of Diseases, 10th Revision
LDL	Low-Density Lipoprotein Cholesterol
NIHSS	National Institutes of Health Stroke Scale
OR	Odds Ratio
RNS	Reactive nitrogen species
ROS	Reactive oxygen species
Se	Sensitivity
Sp	Specificity
TG	Triglycerides
TNFα	Alpha Tumor Necrosis Factor
